# Natural Polyphenols as Modulators of Etoposide Anti-Cancer Activity

**DOI:** 10.3390/ijms22126602

**Published:** 2021-06-20

**Authors:** Magdalena Kluska, Katarzyna Woźniak

**Affiliations:** Department of Molecular Genetics, Faculty of Biology and Environmental Protection, University of Lodz, 90-236 Łódź, Poland; magdalena.kluska@edu.uni.lodz.pl

**Keywords:** polyphenols, etoposide, topoisomerase II

## Abstract

Polyphenols are naturally occurring compounds found in abundance in fruits and vegetables. Their health-promoting properties and their use in the prevention and treatment of many human diseases, including cancer, have been known for years. Many anti-cancer drugs are derived from these natural compounds. Etoposide, which is a semi-synthetic derivative of podophyllotoxin, a non-alkaloid lignan isolated from the dried roots and rhizomes of *Podophyllum peltatum* or *Podophyllum emodi* (Berberidaceae), is an example of such a compound. In this review, we present data on the effects of polyphenols on the anti-cancer activity of etoposide in in vitro and in vivo studies.

## 1. Introduction

Polyphenols are biologically active compounds that are abundant in various parts of plants—fruits, leaves, roots and seeds. They show many health-promoting properties, mainly antioxidant, anti-inflammatory and antibacterial. Recently, there is an increasing interest in polyphenols in the treatment of different pathological states, such as cardiovascular and neurodegenerative diseases and cancer [1,2,3]. Many studies indicate the anti-cancer properties of polyphenols [4,5,6]. Polyphenols exert their potential in anti-cancer therapy via promoting apoptosis and cell senescence, regulating autophagy, and inhibiting proliferation and migration of cancer cells. They can induce cellular stress and catabolism: through an increase in reactive oxygen species (ROS) and a decrease in cellular antioxidants such as glutathione (GSH), interaction with chemotherapy agents and the reduction or reversal of multidrug resistance [7]. Polyphenols can change epigenetic modifications, such as DNA methylation and histone modifications, as well as regulate non-coding miRNAs expression, which modulates gene expression. These epigenetic changes, which induce polyphenols and other phytochemicals, play a crucial role in cancer prevention and therapy [5]. Recent studies indicate that polyphenols inhibit senescence-associated secretory phenotype (SASP) and form an anti-cancer microenvironment to prevent cancer. On the other hand, when tumorigenesis occurs, polyphenols are able to inhibit the cancer by the mechanisms of oncogene-, oxidative stress-, DNA damage response (DDR)-, and endoplasmic reticulum (ER) stress-induced cancer cell senescence. Pro-senescence therapy is a recently proposed anti-cancer strategy and has been shown to effectively inhibit cancer [6].

In the last twenty years, the number of publications on the possibility of using polyphenols in the treatment of cancer has increased significantly [8]. There are several reasons for this increase in interest in polyphenols. On the one hand, it is certainly a disappointment with chemotherapy, which is still one of the basic methods of cancer treatment and, in many cases, is a very effective method. However, it should not be forgotten that chemotherapy carries the risk of often serious side effects, which may lead to the patient’s death. In addition, cancer cells may become resistant to the chemotherapy used. It often happens that chemotherapy does not bring the expected results. Natural substances, such as polyphenols, have a weaker anti-cancer activity compared to classic chemotherapeutic agents. However, studies indicate that polyphenols can act synergistically with drugs, increasing their effectiveness. In addition, some studies indicate that polyphenols are less cytotoxic to normal cells compared to cancer cells. As components of complementary or supportive therapy, they could have a protective and health-promoting effect on the patient’s body [2,4,7,8].

**Figure 1:**
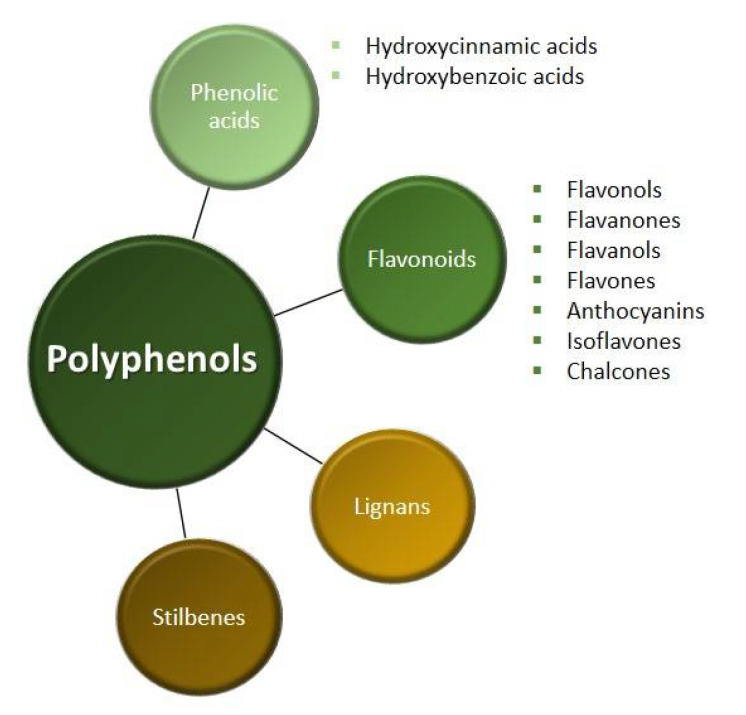
Classification of polyphenols [4,8].

**Figure 2:**
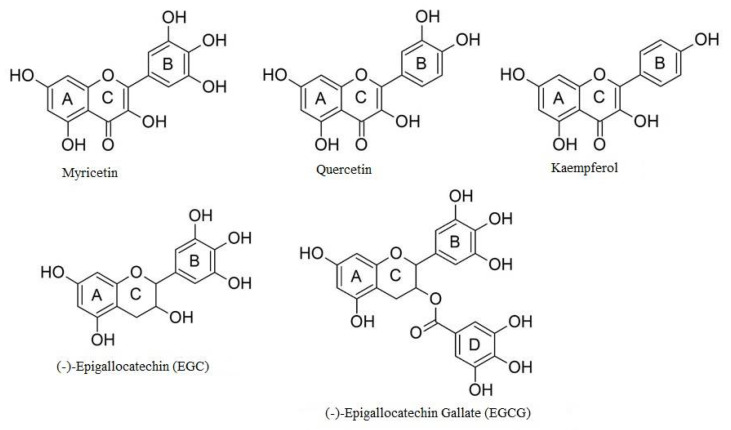
Flavonoids being TopoII poisons.

We divide polyphenols into four groups: phenolic acids, flavonoids, lignans and stilbenes (Figure 1). The largest and best-known group of polyphenolic compounds are flavonoids. They can inhibit the activity of human topoisomerase II (TopoII) by acting as so-called TopoII poisons (Figure 2). TopoII poisons have found application in the treatment of cancer, but they can also contribute to the development of leukemias. Their action is based on covalent or non-covalent bonding with TopoII, thus disturbing its functions [9].

Polyphenols can modulate the action of many anti-cancer drugs through various mechanisms [5,8,10]. In this review, we described the effect of polyphenols on the activity of etoposide, which is one of the anti-cancer drugs derived from natural compounds. We also briefly present the molecular mechanisms responsible for the observed effects of polyphenols on the anti-cancer activity of etoposide in cancer cell lines and in animal models.

## 2. Polyphenols

Polyphenols are the most common antioxidants in the human diet, with a total intake as high as 1 g per day [11]. Polyphenols are compounds derived from phenylalanine, having at least one aromatic ring with one or more hydroxyl groups attached [4]. They are divided into two main groups, flavonoids and non-flavonoids, which depend on the number of benzene rings.

**Figure 3:**
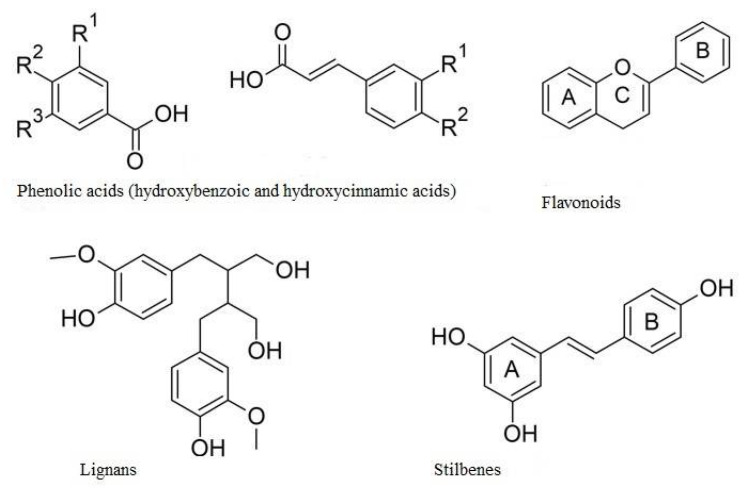
Structures of the four main groups of polyphenols.

Non-flavonoids contain up to two benzene rings and among them we distinguish phenolic acids, phenolic alcohols, stilbenes and lignans (Figure 3). Phenolic acids and phenolic alcohols have a single benzene ring and a carboxylic or hydroxyl terminal functional group, respectively. Among the phenolic acids we distinguish the hydroxybenzoic acids (e.g., ellagic acid and gallic acid) and hydroxycinnamic acids (e.g., ferulic acid and chlorogenic acid). Hydroxybenzoic acids are present in few edible plants while hydroxycinnamic acids are more common in food, especially in coffee. Both stilbenes and lignans have two benzene rings, but stilbenes have a linear and lignans have a branched polymer structure. The most important lignans are sesamin and secoisolariciresinol diglucoside, while the stilbenes are resveratrol, pterostilbene and piceatannol [12].

Flavonoids are made up of more than two benzene rings and they include flavonols, flavones, flavanones, flavanols, anthocyanidins, isoflavones and chalcones. Flavonols are the most abundant flavonoids in foods, but they are present at relatively low concentrations. This group of compounds includes kaempferol, quercetin, myricetin, galangin and isorhamnetin. Flavonols are present in glycosylated forms with an attached sugar moiety, such as glucose or rhamnose. The richest sources of flavonols are onions, broccoli, blueberries, red wine and tea. Flavones available in food are the glycosides of luteolin and apigenin. They are less common than flavonols and can be found in parsley and celery. Flavanones such as naringenin and hesperetin are mainly found in citrus fruits and aromatic plants, such as mint and tomatoes. Flavanols exist as simple monomers (catechins) and as dimers, oligomers and polymers of catechins (proanthocyanidins). Among the flavanols, epicatechin, epigallocatechin, gallocatechin and epigallocatechin gallate are distinguished. These compounds are widespread in medicinal herbs and diet plants, such as tea, apples, berries, cocoa, grapes, wine and legumes. Proanthocyanidins form complexes with salivary proteins and are responsible for the bitterness of chocolate and the astringent character of fruit and beverages. Isoflavones have structural similarities to estrogens that confer their pseudohormonal properties, including the ability to bind to estrogen receptors. For this reason, isoflavones are classified as phytoestrogens. The main source of isoflavones is soya, which contains genistein, daidzein and glycitein [12,13].

## 3. Etoposide

**Figure 4:**
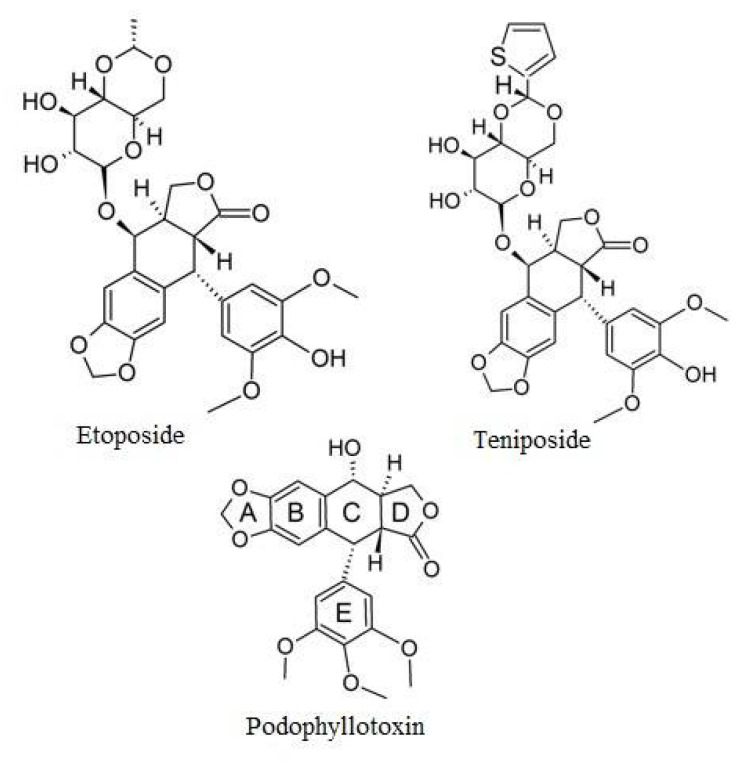
Drugs based on podophyllotoxin.

Etoposide (VP-16, epipodophyllotoxin) is a semi-synthetic derivative of podophyllotoxin, a non-alkaloid lignan isolated from the dried roots and rhizomes of *Podophyllum peltatum* or *Podophyllum emodi* (*Berberidaceae*) (Figure 4). Etoposide was approved for clinical use in the United States of America in 1983 [14]. Although etoposide was synthesized many years ago, it is still widely used in chemotherapy for many types of cancer, including small cell lung carcinoma [15], testicular carcinoma [16], leukemia [17], adrenocortical carcinoma [18], breast cancer [19] or brain tumors [20].

Topoisomerases are essential enzymes involved in relaxation of DNA for transcription and replication, DNA repair and chromatin remodeling. Topoisomerases are grouped into type I (TopoI) and type II (TopoII) based on the number of strand breaks introduced by these enzymes. TopoII exists in two isoforms: TopoIIα, which is active in proliferating cells and plays a role in DNA replication, and TopoIIβ, active in regions of active transcription and expressed in all cells. TopoIIα is overexpressed in cancer cells and is not expressed significantly in quiescent cells [21]. There are several anti-cancer drugs that act as topoisomerase poisons, such as etoposide, doxorubicin and mitoxantrone [22]. The action of etoposide is based on the inhibition of TopoII, which leads to the topoisomerase II-mediated DNA cleavage [23]. The presence of TopoII-cleavage complexes induced by etoposide leads to the formation of single- and double-strand DNA breaks. Moreover, etoposide prevents re-ligation of the double-strand DNA breaks [24]. In addition to topoisomerase, also chromatin can be a target of the drug because etoposide shows a high affinity for chromatin and histones, especially H1 [25]. Moreover, it was shown that etoposide inhibits mitosis, and stops cell division at the S phase or the G2 phase [26]. Etoposide triggers caspase-mediated apoptosis, which mainly occurs through the cytochrome c/caspase 9 pathway [27]. Additionally, etoposide treatment triggers Fas ligand (FasL) binding to its receptor (FasR) on the cell membrane, resulting in formation of the death-inducing signaling complex (DISC). The DISC-binding protein FADD binds to the pro-caspase 8 and promotes its self-cleavage to caspase 8. Then caspase 8 interacts with some effector caspases, such as caspase 3 [24]. Moreover, etoposide is metabolized by cytochrome P450, horseradish peroxidase and tyrosinase to an etoposide phenoxy radical, o-quinone-etoposide. The presence of the 4′-OH in etoposide has been found to be essential for the formation of the radical of this drug, its metabolites as well as the antitumor activity [28].

The low water solubility and poor bioavailability of etoposide, as well as the development of drug resistance, metabolic inactivation and toxic side effects, are major challenges in etoposide-based therapy. The side effects of etoposide include bone marrow suppression, mucositis, cardiotoxicity, nephrotoxicity, hair loss and immunosuppression [26]. An important side effect of etoposide use is the occurrence of acute myelocytic leukemia (t-AML) and treatment-related myelodysplastic syndromes (t-MDS), which often develop into t-AML [24].

## 4. Polyphenols as Poisons of Topoisomerase II

**Figure 5:**
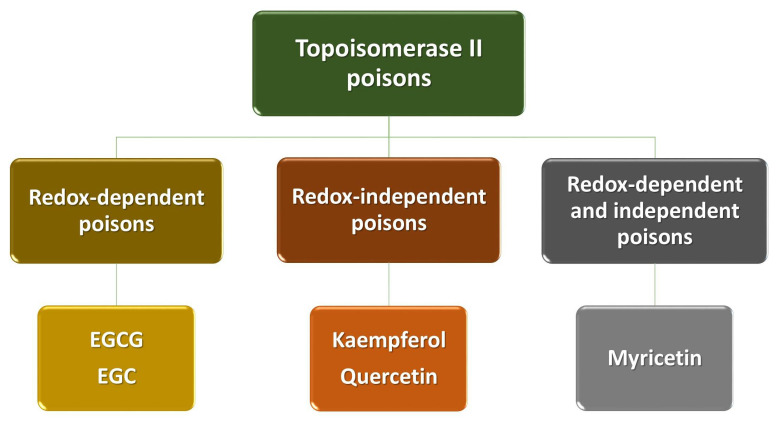
Polyphenols as TopoII poisons [9].

Polyphenols are a diverse and complex group of compounds that are linked to human health. Many of their effects have been attributed to the ability to poison TopoII (i.e., enhance DNA cleavage) (Figure 2). Polyphenols act against the enzyme by at least two different mechanisms. Some compounds are traditional, redox-independent TopoII poisons, interacting with the enzyme in a noncovalent manner. Conversely, others enhance DNA cleavage in a redox-dependent manner, which requires covalent adduction to TopoII. Studies conducted by Bandele and colleagues [9] have shown that polyphenols can be divided into four groups: EGCG and EGC are redox-dependent TopoII poisons; kaempferol and quercetin are traditional poisons; myricetin utilizes both mechanisms; and ECG and EC display no significant activity (Figure 5). On the basis of these findings, a set of rules are proposed that predict the mechanism of bioflavonoid action against TopoII (Table 1). The first rule centers on the B ring. While the C4′-OH is critical for the compound to act as a traditional poison, the addition of -OH groups at C3′ and C5′ increases the redox activity of the B ring and allows the compound to act as a redox-dependent poison. The second rule centers on the C ring. The structure of the C ring in the flavonols is aromatic and planar, and includes a C4-keto group that allows the formation of a proposed pseudo ring with the C5-OH. Disruption of these elements abrogates binding to the enzyme and precludes the ability to function as a traditional TopoII poison [9].

## 5. Polyphenols as Modulators of Etoposide Activity

### 5.1. In Vitro Models

Polyphenols modulate the therapeutic effect of etoposide by increasing its cytotoxicity in many types of cancer cell lines including leukemia [29,30], breast cancer [31], liver cancer [32], cervical cancer [33], colon cancer [34,35], head and neck cancer [36], lymphoma [37,38], osteosarcoma [39], gastric cancer [40,41], glioblastoma [42] and retinoblastoma [43] (Table 2). This activity of polyphenols is associated with an increase in apoptosis and DNA damage, ROS production and cell cycle arrest. Studies carried out on MDA-MB-231 human breast cancer cell line showed that flavonoids, such as kaempferol, fisetin, quercetin, (−)-catechin, genistein, naringenin and cyanidin, inhibited the DNA damage checkpoints and repair pathways. The tested polyphenols inhibited etoposide-induced Chk1 Ser^345^ phosphorylation, leading to disruption of the ATR-Chk1 pathway [44]. Thus, polyphenols can increase the therapeutic effect of chemotherapy by making cancer cells more sensitive to drugs. Studies on HL-60 promyelocytic leukemia cells showed that kaempferol increased the DNA damage induced by etoposide [45]. On the other hand, it was shown that kaempferol increased the sensitivity of HL-60 cells to etoposide but did not increase apoptosis and the ROS level induced by the drug [46]. Interestingly, glycoside derivatives of kaempferol isolated from aerial parts of *Lens culinaris* Medik. may have a different effect on the action of etoposide in HL-60 cells compared to kaempferol. For example, kaempferol 3-*O*-[(6-*O*-E-caffeoyl)-β-d-glucopyranosyl-(1→2)]-β-d-galactopyranoside-7-*O*-β-d-glucuropyranoside reduced the apoptosis and increased the level of free radicals induced by etoposide [46]. Moreover, glycoside derivatives of kaempferol reduced DNA damage induced by etoposide in peripheral blood mononuclear cells (PBMCs) but did not have an impact on DNA damage in HL-60 cells [45].

Curcumin increased the level of apoptotic HL-60 leukemia cells in comparison to cells treated only with etoposide [30]. Moreover, an increase in apoptosis level was associated with enhancement of histone H2AX phosphorylation, which is a marker of DNA damage. Co-treatment with curcumin also increased the apoptosis level in Weri-Rb1 and Y79 retinoblastoma cell lines by upregulating the expression of caspase-3 [43]. Additionally, curcumin decreased the percentage of these cells in the G0/G1 cell cycle phase. In U-87MG glioblastoma cells treated with etoposide, curcumin decreased the expression of p10 and p53 genes and increased the BAX/Bcl-2 ratio [42]. The combination of curcumin and etoposide induced apoptosis of SGC-7901 gastric cancer cells by suppressing nuclear transcription factor NF-κB and NF-κB-regulated anti-apoptotic genes Bcl-2 and Bcl-xL [40]. Co-incubation of LT12 cells derived from BNML rats with curcumin and etoposide leads to an increase in the DNA damage level and apoptosis. An increase in the number of cells arrested in the G2/M phase was also observed [47].

Treatment of the Ramos non-Hodgkin’s lymphoma cell line with EGCG alone leads to the ROS generation, mitochondrial damage and apoptosis induction [38]. Treatment of these cells with etoposide and EGCG synergistically induced apoptosis. Additionally, EGCG sensitized the human breast cancer cells MDA-MB-231 and T-47D to apoptosis induced by etoposide [31]. EGCG binds to the GRP78 protein, leading to inhibition of its ATPase activity and interferes with formation of the anti-apoptotic GRP78-caspase-7 complex. This leads to an increase in etoposide-induced apoptosis and suppresses the number of cells with a transformed phenotype after etoposide treatment. Taken together, these results indicate that EGCG can assist in preventing the drug resistance of cancer cells.

Interestingly, the combination of etoposide and fisetin in osceosarcoma MG-63 and Saos-2 cells induced negative-to-positive interactions on the proliferation, depending on the relative concentrations [39]. Treatment with fisetin and etoposide increased the amount of cells in the G2 phase and decreased the amount of cells in the G1 phase of the cell cycle. Moreover, co-treatment decreased the level of cyclins B1 and E1, which highlighted the anti-proliferative activity of the combination of these compounds. The addition of fisetin to etoposide treatment resulted in obtaining a greater therapeutic effect and allowing the use of lower drug doses.

Gossypol, a polyphenolic aldehyde derived from cotton plants (*Gossypium* spp., *Malvaceae*), may bind to the BH3 binding groove of the anti-apoptotic proteins Bcl-xL and Bcl-2 [48]. Simultaneous incubation of Ramos non-Hodgkin’s lymphoma cells with gossypol and etoposide increased apoptosis in a time-dependent manner via enhancement of cytosolic cytochrome c release and activation of caspase-3 signaling [37].

While etoposide stopped HCT116 colon cancer cells in the G2/M phase of the cell cycle, the combination of etoposide and quercetin restored the course of the cell cycle. Quercetin reduced the level of cyclin B1, a major G2/M regulator. Furthermore, quercetin abrogated the increase in p53 or its targets BAX and p21 levels induced by etoposide [49]. The Mixed Lineage Leukemia gene (MLL) rearrangements induce infant and adult leukemias. It is established that both etoposide and quercetin play a role in the generation of MLL rearrangements [50,51]. Combination of these compounds increased the frequencies of MLL rearrangements in the human hematopoietic stem and progenitor cells CD34+ HSPCs [52].

Simultaneous treatment of SCC25, CAL27 and FaDu head and neck squamous cell carcinoma cell lines with resveratrol (3,4′,5-trihydroxystilbene) and etoposide improved apoptosis and necrosis in comparison to cells treated only with polyphenol or a drug [36]. Likewise, in studies of Hwang and colleagues [34], it was shown that resveratrol enhanced the therapeutic effect of etoposide. Combination treatment enhanced the chemosensitivity of cells through the activation of (AMP)-activated protein kinase (AMPK), increasing ROS production and induction of apoptosis in HT-29 colon cancer cells. Polyphenolic compounds may also find application in the treatment of tumors containing cancer stem cells (CSC), which are resistant to the action of chemotherapeutic agents. Unfortunately, CSC has been identified in most human tumors [53,54]. Ruiz et colleagues demonstrated that the inhibition of RAD51 expression was critical for chemosensitization of CSC to etoposide treatment [33]. Resveratrol is a factor that reduces the expression of RAD51 in CSC derived from HeLa cell cultures. Co-treatment of these cells with etoposide and resveratrol decreased cell viability and induced apoptosis. Similarly, resveratrol inhibited the expression of XRCC1 and enhanced the etoposide-induced cell death and anti-proliferation effect in non-small cell lung cancer cells (NSCLC) [55]. Resveratrol also enhanced the anti-proliferative effects of etoposide in HepG2 and HCT116 cells, but this enhancement was more pronounced in the case of the HCT116 cell line [35]. Pre-treatment of both cell types with resveratrol increased the expression of p53 induced by etoposide and, as with cytotoxicity, the increase in p53 expression was higher in HCT116 cells.

Rhamnetin improved the level of HepG2 cells in the S phase of the cell cycle, which led to an increased etoposide cytotoxic effect and reduction of the etoposide IC_50_ value [32]. Therefore, rhamnetin may find application in lowering the therapeutic doses of etoposide.

On the other hand, some polyphenols did not modulate the etoposide activity. Taurin did not enhance the cytotoxicity induced by etoposide in MCF-7 breast cancer, HepG2 liver cancer, HCT116 colon cancer, U251 glioblastoma and HeLa cervical cancer cell lines [56]. Genistein also had no impact on the cytotoxicity and genotoxicity induced by etoposide in the CEM lymphoblastic leukemia cell line [57].

Moreover, some polyphenols reduced the anti-cancer activity of etoposide. Curcumin decreased the cytotoxicity of etoposide in MCF-7 breast cancer cells, HepG2 liver cancer cells, HCT116 colon cancer cells and HeLa cervical cancer cells [56]. Co-treatment with curcumin and etoposide increased the fraction of MCF-7 cells in the S phase of the cell cycle and increased the fraction of HCT116 and HeLa cells in the G2/M phase. It was proposed that this antagonistic interaction between curcumin and etoposide is due to the cell cycle arrest, which allows time for DNA damage repair and prevents cell death. Another polyphenol, quercetin, can limit the action of etoposide. Quercetin protected HL-60 cells from etoposide by decreasing the level of ROS generated in drug-treated cells [58]. Moreover, quercetin decreased the etoposide induced fraction of late apoptotic cells and the sub-G1 fraction that corresponds to apoptotic cells. Resveratrol, used individually, acts as a SIRT1-activating compound (STACs) and induces apoptosis in Ewing’s sarcoma cells (SK-ES-1, SK-N-MC, WE-68) [60]. Unfortunately, resveratrol prevents Ewing’s sarcoma cells from etoposide-induced cell death. It is worth noting that resveratrol significantly reduces the etoposide-induced expression of p21 in WE-68 cells. Therefore, resveratrol can alter the anti-cancer activity of etoposide by modulating the gene expression.

Interesting results were obtained by Mahbub and colleagues [29] based on research on two lymphoid (CCRF-CEM and Jurkat) and two myeloid (THP-1 and KG1a) cell lines, and on two non-tumor control haemopoietic stem cell lines (HSCs) (CD133+ and CD34+). These results were additionally thoroughly discussed by them [61]. Five polyphenols have been selected based on previous results relating to their apoptotic effect on leukemia cells. Quercetin, apigenin, emodin, rhein and *cis*-stilbene were combined with anti-cancer drugs doxorubicin or etoposide. Then, several parameters describing the state of the cells after treatment with the combination of drugs and polyphenols were examined. Results showed that etoposide in combination with the tested polyphenols synergistically reduced the ATP levels, induced apoptosis and increased the S and/or G2/M phase cell cycle arrest in both lymphoid leukemia cell lines. Moreover, a reduction in the GSH level in lymphoid cell lines was observed after treatment with etoposide and all the tested polyphenols. In contrast, in the myeloid cell lines, co-treatment induced different kinds of responses. Emodin, rhein and *cis*-stilbene inhibited apoptosis, increased the ATP level and enhanced the GSH level in cells treated with etoposide, while quercetin and apigenin increased apoptotic cell death and decreased the GSH level. All tested polyphenols had a protective effect on the normal HCS cells manifested through an increase in cell proliferation and a decrease in apoptosis. The basal level of glutathione (GSH) was higher in lymphoid leukemia cell lines than in myeloid leukemia and non-tumor control HSCs. It was established that a reduction in the GSH level is crucial for normal cells to undergo apoptosis independent of ROS formation [62]. These results lead to the statement that an increase in the anti-cancer effect of co-treatment with polyphenols and etoposide is associated with a decrease in the amount of GSH.

### 5.2. In Vivo Models

The modulatory effect of polyphenols on etoposide activity was also described in in vivo models (Table 3). Jiang and coworkers conducted the in vivo studies on BALB/c nude mice bearing SGC7901 cells xenografts to examine whether the co-delivery of etoposide and curcumin with one nanoparticle can result in synergistic effects of both drugs. They administrated the tested solutions through the tail vein of the mice, once every 3 days, and the tumor volume of each mouse was measured with a digital caliper every 3 days. After the study, they found that the mice bearing gastric tumor SGC7901 cells injected with a combination of etoposide and curcumin have a smaller tumor volume than mice injected only with etoposide [41]. These results suggest that a combination of etoposide and curcumin, which is a known effective NF-κB inhibitor, may improve the therapeutic efficacy in cancer treatments. In Brown Norway rats with transplantable acute promyelocytic leukemia (BNML), curcumin also increased the anti-leukemic activity of etoposide [30]. Rats were infused with leukemic cells and then treated with curcumin for 23 days (100–200 mg/kg). Etoposide (50 mg/kg) was administered for the final 3 days of the experiment. It was observed that etoposide alone efficiently reduced the progression of leukemia in rats. In addition, curcumin itself, administered to animals at a dose of 200 mg/kg, showed a similar antitumor effect as etoposide. Moreover, the rats that received 200 mg/kg curcumin followed by administration of etoposide had a 1.57-fold tumor reduction and a greater reduction in the spleen weight in comparison to animals that received the only etoposide. Additionally, the proportion of leukemic cells and healthy B-cells was corrected. Other studies carried out on BNML rats revealed that curcumin significantly decreased the number of leukemic promyelocytes in the bone marrow of BNML rats compared to the leukemic control [63]. Co-treatment with curcumin and etoposide decreased the number of promyelocytes to the normal values occurring in healthy individuals. It also leads to an increase in the percentage of the normal precursors of granulocytes and erythrocytes. In summary, these studies indicate that curcumin may protect normal myeloid precursors against the cytotoxic effects of chemotherapy based on etoposide.

Epicatechin exhibits a similar modulating effect on etoposide-based chemotherapy in BNML rats as curcumin. Treatment with epicatechin (40 mg/kg) increased the apoptotic effect of etoposide (50 mg/kg) both in the spleen and the bone marrow in comparison to animals who received the only etoposide in vivo [64]. It was discovered that BNML rats given epicatechin at a dose of 40 mg/kg had a 62% decrease in the number of promyelocytes when compared to the control. Etoposide administration caused a decrease in the percentage of promyelocytes by 81% and co-treatment with epicatechin and etoposide resulted in a further decrease to 92% in the number of promyelocytes in comparison to the control. Moreover, epicatechin increased the oxidative stress induced by etoposide by reduction of superoxide dismutase (SOD) activity measured in liver homogenates. In rats co-treated with epicatechin and etoposide, a decrease in the SOD activity was observed compared to animals receiving etoposide only. Additionally, examination of the malondialdehyde concentration in rat liver and the ferric ion-reducing ability of the plasma levels showed that epicatechin synergistically increases the oxidative stress induced by etoposide in BNML rats [64]. All these results indicated that epicatechin modulates the anti-cancer activity of etoposide in BNML rats by increasing oxidative stress.

Studies on the Brown Norway rat promyelocytic leukemia LT12 cell line and bone marrow cells of BN/CrlCmd rats were carried out to investigate the influence of quercetin on myelosuppression and oxidative DNA damage caused by etoposide [59]. Quercetin was administrated at a dose of 100 mg/kg for 14 consecutive days and etoposide was administered intraperitoneally for three consecutive days at a dose of 50 mg/kg. It was shown, using a comet assay, that quercetin reduced the oxidative DNA damage caused by etoposide in rat bone marrow cells. In the case of the LT12 cell line, pre-incubation with quercetin followed by co-treatment with etoposide also reduced the level of oxidative DNA damage in comparison to cells treated only with etoposide. Furthermore, administration of quercetin prevented the decrease in the percentage of myeloid precursors and erythroid nucleated cells caused by etoposide. These results suggest that quercetin may protect bone marrow cells from the harmful effects of chemotherapy based on etoposide.

The data collected in Table 2 and Table 3 clearly show that most polyphenols act synergistically with etoposide, increasing its antitumor activity. However, it seems that this expected effect of polyphenols on cancer cells is due to their specific concentrations and the concentration of etoposide. Achieving an appropriate concentration ratio in a cell culture is not a problem; however, it can be very difficult or impossible to achieve in vivo. Another limiting factor in the use of polyphenols in etoposide therapy may be the antioxidant activity of polyphenols, which may reduce the pro-oxidative effect of the drug.

## 6. Conclusions

More than 25% of the drugs used during the last 20 years are directly derived from plants, while the other 25% are chemically altered natural products [65]. One of these types of anti-cancer drugs is etoposide. The studies described in this review indicate that polyphenols may modulate the anti-cancer activity of etoposide in a variety of cancer cells. Most polyphenols act synergistically with etoposide, increasing cell apoptosis, DNA damage and arresting the cell cycle. It should be emphasized that most of the studies conducted so far have been performed in vitro. The potential use of polyphenols in etoposide chemotherapy requires detailed in vivo research preceded by the development of effective methods of obtaining and administration of polyphenolic drugs.

## Figures and Tables

**Table 1:** Polyphenols as TopoII poisons [9].

Polyphenol	Proposed Mechanisms of Action
EGCG EGC	Presence of -OH groups at C3′ and C5′ increases the redox activity of the B ring and allows the compound as a redox-dependent poison.
Kaempferol Quercetin	Presence of -OH groups at C4′ of the B ring which makes the compound acts like a traditional, redox-independent poison. Aromatic and planar structure of the C ring with a C4-keto group that allows the formation of a proposed pseudo ring with the C5-OH.
Myricetin	Presence of -OH groups at C3′ and C5′ increases the redox activity of the B ring and allows the compound as a redox-dependent poison. Presence of -OH groups at C4′ of the B ring which makes the compound acts like a traditional, redox-independent poison. Aromatic and planar structure of the C ring with a C4-keto group that allows the formation of a proposed pseudo ring with the C5-OH.

**Table 2:** Effect of polyphenols on the anti-cancer activity of etoposide—in vitro studies.

Polyphenol	In Vitro Model	Dose of Polyphenol	Dose of Etoposide	Interaction with Etoposide	Ref.
Apigenin	CCRF-CEM	LSD	LSD	ATP level ↑; caspase-3 and 9 activity ↑; level of cells in S and G2/M phase of cell cycle ↑; glutathione level ↓; γH2AX foci ↑	[29]
	Jurkat	LSD	LSD	ATP level ↑; caspase-3 and 9 activity ↑; level of cells in S phase of cell cycle ↑; glutathione level ↓; γH2AX foci ↑	[29]
	KG-1a	LSD	LSD	ATP level ↑; caspase-3 and 9 activity ↑; level of cells in S and G2/M phase of cell cycle ↑; glutathione level ↓; γH2AX foci ↑;	[29]
	THP-1	LSD	LSD	ATP level ↓; caspase-3 and 9 activity ↑; level of cells in S and G2/M phase of cell cycle ↑; glutathione level ↓; γH2AX foci ↑	[29]
Catechin	MDA-MB-231	10–40 µM	1 µM	inhibition of etoposide-induced Chk1 Ser^345^ phosphorylation	[44]
Curcumin	HL-60	20 µM	3–10 µM	apoptosis ↑; phosphorylation of the histone H2AX induced by etoposide ↑; ROS generation ↑	[30]
	SGC7901	1 mg	5 mg	cytotoxicity induced by etoposide ↑	[41]
	Weri-Rb1 and Y79	5–10 µM	0.1–20 µg/mL	etoposide-induced cytotoxicity ↑; level of apoptotic cells ↑; caspase 3 activity ↑; level of the cells in the G0/G1 phase of the cell cycle ↓	[43]
	LT12	1–20 µM	1–40 µM	level of cells arrested in the G2/M phase ↑; DNA damage ↑; number of apoptotic cells ↑	[47]
	MCF-7, HepG2, HCT116, HeLa	10 µg/mL	1 µg/mL	cytotoxicity of etoposide ↓; level of MCF-7 cells in S phase of cell cycle ↑; level of HCT116 and HeLa cells in the G2/M phase ↑;	[56]
	U-87MG	37.33 µg/mL (IC_50_)	6.5 µg/mL	cytotoxicity induced by etoposide ↑; BAX/Bcl-2 ratio ↑; expression of *p10* and *p53* ↓	[42]
	SGC7901	10–160 µM	2–200 µM	etoposide-induced cytotoxicity ↑; phosphorylation of IκBα ↓; level of apoptotic cells ↑; Bcl-2 and Bcl-xL expression ↓; attenuated the activation of NF-κB	[40]
Cyanidin	MDA-MB-231	10–40 μM	1 µM	inhibition of etoposide-induced Chk1 Ser^345^ phosphorylation	[44]
EGCG	MDA-MB-231	10–40 μM	1 µM	inhibition of etoposide-induced Chk1 Ser^345^ phosphorylation	[44]
	Ramos	7.5 µM	0.02 µg/mL	apoptosis induced by etoposide ↑	[38]
	MDA-MB-231 and T-47D	10 µM	0.1 µM	interferes with the formation of the anti-apoptotic GRP78-caspase-7 complex, which leads to an increase etoposide-induced apoptosis; suppresses the transformed phenotype of breast cancer cells treated with etoposide	[31]
Emodin	CCRF-CEM	LSD	LSD	ATP level ↓; caspase-3 and 9 activity ↑; level of cells in S phase of cell cycle ↑; glutathione level ↓; γH2AX foci ↑	[29]
	Jurkat	LSD	LSD	ATP level ↓; caspase-3 and 9 activity ↑; level of cells in S and G2/M phase of cell cycle ↑; glutathione level ↓; γH2AX foci ↑	[29]
	KG-1a	LSD	LSD	caspase-9 activity ↑	[29]
	THP-1	LSD	LSD	caspase-9 activity ↑	[29]
Fisetin	MDA-MB-231	10–40 μM	1 µM	inhibition of etoposide-induced Chk1 Ser^345^ phosphorylation	[44]
	MG-63 and Saos-2	5–150 µM	0.5–10 µM	shows negative-to-positive interactions on the inhibition of cell proliferation depending on the relative concentrations; level of cells in G2-phase of the cell cycle ↑; cells in G1-phase ↓; levels of cyclins B1 and E1 ↓	[39]
Gossypol	Ramos	12 µM	20 µM	apoptosis in a time-dependent manner via activation of caspase-3 signaling ↑; enhances cytosolic cytochrome c release ↑	[37]
Genistein	MDA-MB-231	10–40 μM	1 µM	inhibition of etoposide-induced Chk1 Ser^345^ phosphorylation	[44]
	CEM	50 µM	0–200 µM	no impact on the cytotoxicity and genotoxicity induced by etoposide	[57]
Kaempferol	MDA-MB-231	10–40 μM	1 µM	inhibition of etoposide-induced Chk1 Ser^345^ phosphorylation	[44]
	HL-60	10–50 µg/mL	1 µM	DNA damage induced by etoposide ↑	[45]
	HL-60	10–50 µg/mL	1–10 µM	sensitivity of cells to etoposide ↑; ROS generation ↓	[46]
Naringenin	MDA-MB-231	10–40 μM	1 µM	inhibition of etoposide-induced Chk1 Ser^345^ phosphorylation	[44]
Quercetin	MDA-MB-231	10–40 μM	1 µM	inhibition of etoposide-induced Chk1 Ser^345^ phosphorylation	[44]
	HL-60	0.5–100 µM	1–10 µM	ROS generation ↓; apoptosis ↓	[58]
	LT12	1–20 µM	5 µM	oxidative DNA damage ↓	[59]
	HCT116	50 µM	50 µM	cyclin B1 level ↓; abrogates the increase in levels of p53 or its targets BAX and p21 induced by etoposide	[49]
	HSPCs	50 µM	10 µM	frequencies of *MLL* rearrangements in human HSPCs ↑	[52]
	CCRF-CEM	LSD	LSD	ATP level ↓; caspase-3 and 9 activity ↑; level of cells in S and G2/M phase of cell cycle ↑; glutathione level ↓; γH2AX foci ↑	[29]
	Jurkat	LSD	LSD	ATP level ↓; caspase-3 and 9 activity ↑; level of cells in G2/M phase of cell cycle ↑; glutathione level ↓; γH2AX foci ↑	[29]
	KG-1a	LSD	LSD	ATP level ↓; caspase-3 and 9 activity ↑; glutathione level ↓; γH2AX foci ↑	[29]
	THP-1	LSD	LSD	ATP level ↓; caspase-3 and 9 activity ↑; level of cells in S and G2/M phase of cell cycle ↑; glutathione level ↓; γH2AX foci ↑	[29]
Resveratrol	WE-68, SK-ES-1 and SK-N-MC	5–10 µM	0.1–1 µM	etoposide-induced *p21* expression in WE-68 cells ↓; etoposide-induced cell death ↓	[60]
	SCC25, CAL27 and FaDu	40 µM	10 µM	etoposide-induced apoptosis ↑	[36]
	HepG2, HCT-116	12.5–100 µM	1–10 µM	etoposide-induced p53 expression ↑; anti-proliferative effects of etoposide ↑	[35]
	HT-29	50–400 µM	100–500 µM	cell death induced by etoposide ↑; ROS generation ↑; chemosensitivity of cells ↑; AMPK ↑	[34]
	Cancer stem cells (CSC) from HeLa	137 µM	5.8 µg/mL	sensitizes cervical CSC cells to etoposide treatment by RAD51 inhibition	[33]
Rhamnetin	HepG2	3 µM	120 nM	level of cells in S phase of cell cycle ↑; IC_50_ value of etoposide ↓	[32]
Rhein	CCRF-CEM	LSD	LSD	ATP level ↓; caspase-3 and 9 activity ↑; level of cells in G2/M phase of cell cycle ↑; glutathione level ↓; γH2AX foci ↑	[29]
	Jurkat	LSD	LSD	ATP level ↓; caspase-3 and 9 activity ↑; level of cells in G2/M phase of cell cycle ↑; glutathione level ↓; γH2AX foci ↑	[29]
	KG-1a	LSD	LSD	caspase-9 activity ↑; glutathione level ↑	[29]
	THP-1	LSD	LSD	caspase-9 activity ↑; glutathione level ↑	[29]
*cis*-Stilbene	CCRF-CEM	LSD	LSD	ATP level ↓; caspase-3 and 9 activity ↑; level of cells in S phase of cell cycle ↑; glutathione level ↓; γH2AX foci ↑	[29]
	Jurkat	LSD	LSD	ATP level ↓; caspase-3 and 9 activity ↑; level of cells in G2/M phase of cell cycle ↑; glutathione level ↓; γH2AX foci ↑	[29]
	KG-1a	LSD	LSD	caspase-9 activity ↑; glutathione level ↑	[29]
	THP-1	LSD	LSD	caspase-9 activity ↑; glutathione level ↑	[29]
Taurin	MCF-7, HepG2, U251, HeLaand HCT116	10–50 µg/mL	1 µg/mL	no effect on etoposide cytotoxicity	[56]

↑—increase; ↓—decrease; LSD—lowest significant dose.

**Table 3:** Effect of polyphenols on the anti-cancer activity of etoposide—in vivo studies.

Polyphenol	In Vivo Model	Dose of Polyphenol	Dose of Etoposide	Interaction with Etoposide	Ref.
Curcumin	Brown Norway rats with acute myeloid leukemia (BNML)	100 and 200 mg/kg	50 mg/kg	enhances the tumor reduction and induces apoptosis of BNML cells more efficiently than etoposide alone	[30]
	BALB/c mice bearing SGC7901 cells xenografts	1 mg	5 mg	decreases tumor volume in comparison to mice treated only with etoposide	[41]
	BNML rats	200 mg/kg	50 mg/kg	decreases the number of promyelocytes to the normal values occurring in healthy individuals; increases the percentage of the normal precursors of granulocytes and erythrocytes	[63]
(−)-Epicatechin	Brown Norway rats with acute myeloid leukemia (BNML)	40 mg/kg	50 mg/kg	increases the in vivo apoptotic effect of etoposide; increases the oxidative stress induced by etoposide by a decrease in SOD activity	[64]
Quercetin	Bone marrow cells from BN/CrlCmd rats	100 mg/kg	50 mg/kg	reduces oxidative DNA damage; protects against a decrease in the percentage of myeloid precursors and erythroid nucleated cells caused by etoposide	[59]

## Abbreviations

AMPK	(AMP)-activated protein kinase
BNML	Brown Norway Acute Myeloid Leukaemia
CSC	cancer stem cells
DISC	death-inducing signaling complex
EC	(−)-epicatechin
ECG	(−)-epicatechin gallate
EGC	(−)-epigallocatechin
EGCG	(−)-epigallocatechin gallate
FasL	Fas ligand
FasR	Fas receptor
GSH	glutathione
HSCs	haemopoietic stem cell lines
LSD	lowest significant dose
MLL	Mixed Lineage Leukemia gene
NSCLC	non-small cell lung cancer cells
ROS	reactive oxygen species
SOD	superoxide dismutase
STACs	SIRT1-activating compounds
t-AML	acute myelocytic leukemia
t-MDS	treatment-related myelodysplastic syndromes
TopoI	topoisomerase I
TopoII	topoisomerase II
